# Contaminants in an Over-the-Counter Medication and Their Effect on Rheumatoid Arthritis Diagnosis: A Case Report

**DOI:** 10.7759/cureus.104494

**Published:** 2026-03-01

**Authors:** Arzoo Manandhar, Lakshmi Sadasivam

**Affiliations:** 1 Internal Medicine, University of California Los Angeles, David Geffen School of Medicine, Los Angeles, USA; 2 Medicine, University of California Los Angeles, David Geffen School of Medicine, Los Angeles, USA

**Keywords:** adrenal insufficiency, ak forte, artri king, contaminants, dietary and herbal supplements, herbal supplements, iatrogenic cushing, iatrogenic cushing’s syndrome, medication causing adrenal insufficiency, unregulated supplements

## Abstract

Rheumatoid arthritis (RA) is a chronic autoimmune disease. Early diagnosis and intervention are critical, and contaminated medications can complicate or obscure the diagnosis of autoimmune diseases. Herein, we report a case of a 38-year-old man who presented with two to three months of worsening arthralgias and myalgias. He reported using AK Forte, a natural supplement from Mexico that was recalled by the FDA for contamination with diclofenac, dexamethasone, and methocarbamol. AK Forte is a popular supplement used in Latin America, especially amongst manual laborers and other physically demanding job workers, and is easily accessible over the counter. The patient also reported Cushingoid symptoms such as weight gain, facial puffiness, and stretch marks. Upon abruptly discontinuing AK Forte, the patient presented to the emergency department with worsening arthralgias, myalgias, intermittent fever, and tachycardia. Despite being on a supplement contaminated with steroids, the patient did not present with symptoms of adrenal insufficiency throughout his hospital course. After ruling out infectious causes, the patient was diagnosed with persistent sinus tachycardia associated with high RA inflammatory activity. This case demonstrates the importance of obtaining a thorough medication history, including over-the-counter drugs, vitamins, and herbal supplements. This case also highlights the risks associated with unregulated supplements, especially those sold outside the United States, as they may not adhere to FDA standards and may be contaminated with pharmacologically active ingredients, affecting their safety, efficacy, and quality.

## Introduction

Rheumatoid arthritis (RA) is a chronic autoimmune disease characterized by inflammatory polyarthritis, which can cause severe bone and cartilage damage if untreated [1]. Early diagnosis and intervention are key in preventing systemic complications and damage to joints. Contaminated medications, such as AK Forte, complicate or obscure the diagnosis of autoimmune diseases like RA. Supplemental health products and medications, like AK Forte, from other countries often enter the United States (US) through unregulated vendors, online pharmacies, or personal importation, resulting in less oversight by governmental agencies. Patients may obtain these medications for many reasons: lack of access to healthcare, financial savings, cultural familiarity with foreign products, or perceiving them as more “natural” and “safer”. However, many medications sold outside the US do not adhere to FDA standards and may be adulterated with undeclared active ingredients or contaminants that may be harmful, delay diagnosis of diseases, or interact with other treatments. AK Forte is an over-the-counter (OTC) supplement marketed for pain relief and imported from Mexico. In April 2023, the FDA recalled AK Forte for contamination with pharmacologically active substances, including diclofenac, dexamethasone, and methocarbamol [2]. This report explores a case of a patient with a delayed diagnosis of RA and iatrogenic Cushingoid symptoms as a result of taking an undeclared supplement with contaminants.

## Case presentation

A 38-year-old Spanish-speaking man with no significant medical history presented with intermittent fever, persistent tachycardia, and worsening arthralgias and myalgias in October 2024. Upon further questioning, the patient stated his pain was usually alleviated by an OTC medication from Mexico called AK Forte, also known as Artri Ajo King. In January 2024, the patient started taking AK Forte. While taking the medication, his pain was masked, and he developed cushingoid-like features such as facial rounding and a 20-pound weight gain since February 2024. Upon abruptly discontinuing AK Forte, the patient reported having trouble walking and tingling in his fingers. He presented to the emergency department in October 2024 with significantly worsening arthralgias, myalgias, fever, and weakness. After consulting with the Infectious Disease and Endocrinology team, we considered starting a hydrocortisone taper until the HPA (hypothalamic-pituitary-adrenal) axis recovered to avoid the possibility of adrenal insufficiency. However, despite the abrupt cessation of AK Forte, the patient did not present with any symptoms of adrenal insufficiency during his hospital course. On physical examination, he exhibited Cushingoid features such as facial roundness, central obesity, and abdominal, inguinal, axillary, and medial thigh striae. The patient had diffuse joint tenderness and swelling in the fingers, wrists, knees, and toes, and tenderness to palpation of paraspinal muscles and spinous processes. He was persistently tachycardic (110-140 beats per minute), hypertensive, and had intermittent fevers throughout his hospital stay. Given his intermittent fevers, tachycardia, and leukocytosis, further workup was done to rule out infectious, cardiac, and thyroid causes. Following consultation with Rheumatology, the patient was diagnosed with seropositive RA with laboratory results demonstrating an elevated erythrocyte sedimentation rate (ESR) and C-reactive protein (CRP) level and positive rheumatoid factor (RF) and anti-cyclic citrullinated peptide (CCP) antibodies (Table 1).

**Table 1:** Laboratory results during admission This table shows the patient’s laboratory data during hospitalization, indicative of high rheumatoid arthritis activity that had been masked for months by use of over-the-counter AK Forte (Artri Ajo King). Though the patient had clinical signs indicative of steroid use, there was no adrenal suppression noted in labs. H = high, L = low, WBC = white blood cell, Hgb = hemoglobin, CRP = C-reactive protein, ESR = erythrocyte sedimentation rate, RF = rheumatoid factor, anti-CCP= anti-cyclic citrullinated peptide, ACTH plasma-SO = adrenocorticotropic hormone plasma send out

Lab	Value	Result date	Reference values
WBC	(H) 13.0 K/cumm	10/23/24	4.5-10.0 K/cumm
Hgb	(L) 11.6 g/dL	10/23/24	13.5-16.5 g/dL
CRP	(H) 76.7 mg/L	10/23/24	0-7 mg/L
ESR	(H) 32 mm/hr	10/23/24	0-22 mm/hr
RF	(H)733 IU/mL	10/24/24	≤13 IU/mL
Anti-CCP	(H) 398 U/mL	10/24/24	≤16 IU/mL
Ferritin	(H) 281 ng/mL	10/25/24	22-275 ng/mL
Cortisol, random	19.0 mcg/dL	10/27/24	10-20 mcg/dL
ACTH, plasma	23 pg/mL	10/27/24	6-50 pg/mL

AK Forte was discontinued, and the patient was started on diclofenac and given IV Solu-Medrol 30 mg every 12 hours, followed by a prednisone taper as an outpatient. At the end of his hospital course, the patient was pain-free and was counseled regarding OTC drug usage. At discharge, methotrexate and folic acid supplementation were initiated with a plan to titrate, as an outpatient under close Rheumatology follow-up.

## Discussion

AK Forte or Artri King is marketed as a natural product from Mexico, used by many for pain relief. In our case, upon further inspection of the patient’s bottle, the product label listed glucosamine, chondroitin, collagen, vitamin C, turmeric, nettle, omega 3, and methylsulfonylmethane as ingredients. However, a 2023 FDA analysis demonstrated the product contained undisclosed pharmacologically active agents, including diclofenac, dexamethasone, and methocarbamol [2]. AK Forte contamination introduced exogenous pharmacologic agents with the potential to mask or exacerbate RA symptoms and cause other side effects. Diclofenac can transiently suppress inflammation, delaying diagnosis. Chronic, low-dose exposure to a glucocorticoid like dexamethasone can mask RA symptoms.

In addition, the FDA has also advised consumers not to purchase or use AK Forte. In September 2024, the FDA tested AK Forte sold by C&A Naturistics, National City, CA, and found the product contained acetaminophen and diclofenac [2]. Furthermore, in October 2024, C&A Naturistics recalled all AK Forte tablets after the FDA found the product to be tainted with diclofenac, dexamethasone, and methocarbamol [3].

Adulteration of supplemental medication used for joint, bone, and muscle conditions is not uncommon. In fact, AK Forte, previously labeled as Artri King and Artri Ajo King, was recalled by the FDA in April 2022 for containing unlabeled diclofenac and dexamethasone [3,4]. The FDA reported that the products sold under variations of the names Artri (such as Artri Ajo King, Artri King, Artri King Forte) or Ortiga (such as Ortiga Mas Ajo Rey, Ortiga Mas Ajo Rey Extra Forte) contained unlabeled active ingredients that may cause dangerous health outcomes such as adrenal insufficiency, increased risk of cardiovascular events, gastrointestinal bleeds, and hepatotoxicity [5].

A study by Culler et al. reported adverse endocrinologic outcomes observed among preoperative patients endorsing the use of Artri King at an orthopedic surgery clinic at a county safety net hospital in Los Angeles [6]. Out of 13 patients with an average duration of use of 10.2 months, 9 patients demonstrated Artri King-induced adrenal insufficiency and iatrogenic Cushing syndrome, while one patient developed new-onset diabetes while taking supplements [6]. This highlights the importance of clinician awareness of documented cases of adverse events stemming from undeclared active pharmaceutical ingredients.

## Conclusions

To conclude, the patient’s presentation highlights the diagnostic challenges posed by contaminants in innocuous-sounding medications and the importance of obtaining a detailed medication history, especially in patient populations from Mexico. It is imperative to take a multidisciplinary approach in unmasking these wolves in sheep’s clothing - potential environmental or pharmacologic contributors to atypical presentations of autoimmune diseases like RA. Physicians should remain vigilant about drug recalls and counsel patients on the associated risks of non-regulated medications in a culturally sensitive manner. Clinical awareness, patient education on medication safety, and routine screening of ingredients in patients who endorse supplement use are essential for patient safety, timely diagnosis, and management of disease.
